# An Investigation of the Compressive Behavior of Polymer Electrode Membrane Fuel Cell’s Gas Diffusion Layers under Different Temperatures

**DOI:** 10.3390/polym10090971

**Published:** 2018-09-01

**Authors:** Yanqin Chen, Chao Jiang, Chongdu Cho

**Affiliations:** Department of Mechanical Engineering, Inha University, Incheon 22212, Korea; chenyanqin@inha.edu (Y.C.); chiaojiang@inha.edu (C.J.)

**Keywords:** gas diffusion layer, polymer electrode membrane fuel cell, compressive characteristics, thermo-mechanics, microstructure

## Abstract

In this paper, a commercial gas diffusion layer is used, to quantitatively study the correlation between its compressive characteristics and its operating temperature. In polymer electrode membrane fuel cells, the gas diffusion layer plays a vital role in the membrane electrode assembly, over a wide range of operating temperatures. Therefore, understanding the thermo-mechanical performance of gas diffusion layers is crucial to design fuel cells. In this research, a series of compressive tests were conducted on a commercial gas diffusion layer, at three different temperatures. Additionally, a microscopical investigation was carried out with the help of a scanning electron microscope, to study the evolution and development of the microstructural damages in the gas diffusion layers which is caused by the thermo-mechanical load. From the obtained results, it could be concluded that the compressive stiffness of the commercial gas diffusion layer depends, to a great extent, on its operational temperature.

## 1. Introduction

Polymer Electrode Membrane Fuel Cells (PEMFCs) play an important role in automotive power supplies due to their low emission, noiseless and environment-friendly operation, and high efficiency. The PEMFC consists of a thin film membrane electrode assembly (MEA), bipolar plates (BPPs), a polymer electrolyte membrane (PEM), a catalyst and the gas diffusion layers (GDLs) [1]. After spraying a mixture of catalyst, carbon, and electrode, on the solid electrolyte membrane, the GDL can be squeezed from both sides, at a high temperature, to protect the interior of the cell and enable it to function as an electrode. Among the fuel cell components, GDLs have a direct effect on the performance of PEMFC [2]. GDLs have a stronger, and a more structured, support for MEA, and have an excellent electrical conductivity. Furthermore, GDLs have porous structures which can effectively transport hydrogen and oxygen. In addition, a GDL is coated with Teflon, so it does not easily get wet and is able to deal with water [3]. As to its mechanical role in fuel cells, the GDL mainly withstands the compressive force. Generally, the cross-section of a GDL is a three-layer structure comprising a substrate, a microporous layer (MPL), and a permeable layer in between. The substrate is a polymer supported by carbon fibers [4,5], carbon felt, carbon cloth [6] or metal foam [7,8] and filled with resins [9,10]. The MPL is usually coated with polytetrafluoroethylene (PTFE).

Recently, research on GDL has attracted much attention in the field of fuel cell development. The main categories of the research are as follows: mechanical compression behavior [11], pressure distribution [12], electrical properties [13,14], and material structural integrity [15,16]. Faydi et al. [11] studied the dynamic compression coefficients of GDLs, over a temperature range from 25 °C to 400 °C, taking into account the expected vibration effects of fuel cell load conditions. They found that the dynamic compression factor of GDLs linearly increases up to the maximum temperature of 280 °C, after which, it then starts to decrease. Radhakrishnan and Haridoss [14,17] reported that GDLs can withstand repeated compressive loads. These loads influence the GDLs structural characteristics such as its surface morphology, roughness, and its pore size. Escribano et al. [18] tested the compressive properties of GDLs and concluded that the durability of the MEA depends on the stress distribution in GDLs. There is also a theoretical study on the compression analysis model of GDL [19,20]. Nevertheless, the temperature range in which PEMFC fuel cells operate is, at least −20–90 °C, but most of the studies above ignore the effect of temperature on the compression characteristics of GDL. Therefore, although there have been many studies on the compression behavior and physical properties of GDLs, the data are not sufficient to understand the actual conditions under which the GDL operates, since in most studies the compression characteristics of GDLs are assumed to be same regardless of the operating temperature of fuel cells. Hence, the present research investigated the effects of fuel cell operating temperature on GDL’s compressive characteristics.

## 2. Experimental Section

### 2.1. Experimental Setup

A commercial GDL was used as the specimen in this study. Some properties of the specimen are listed in Table 1.

**Figure 1:**
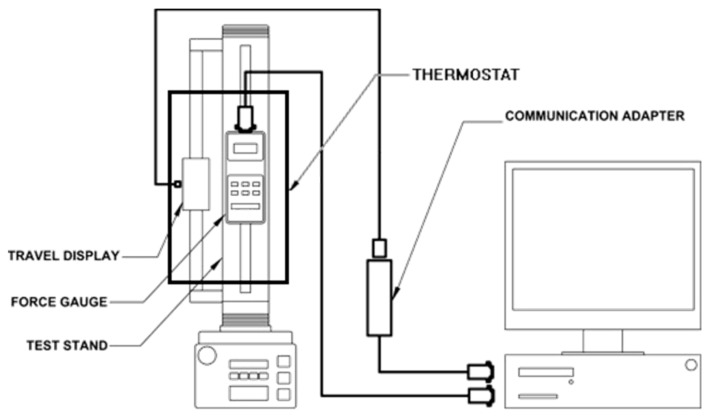
Material tester BS-205 with an environmental chamber.

Considering the influence of the starting temperature of a car parked for a long time at ambient temperature, the operating temperature of most PEMFCs is expected to be within −20 °C to 90 °C [21,22]. During its operation, the GDL is subjected to a mechanical compressive load of fuel cell stacks, in the MEA. In this study, we investigated how the operating temperature affected the compressive characteristics of the commercial GDL. Generally, the relative humidity (RH) range of a fuel cell is between 10% and 100%. As the temperature rises under the test conditions, it becomes more difficult to control the RH, as compared to the temperature. In this experiment, we set the RH to about 30%, in the chamber, and maintained it at a constant level to minimize the effect of humidity on the compressive behavior of the GDL. Compression tests were performed using a Material Tester BS-205 (C & FO Engineering, Osan, Korea) with an environmental chamber, as shown in Figure 1. The specifications of the tester are listed in Table 2. The environmental chamber in Figure 1 can control temperature and humidity, but it does not have a freezing function. Even though the fuel cell starts to operate at cold ambient temperatures, it is expected that the temperature of the GDL rises to 20–90 °C. As the prime objective of this research was to quantitatively confirm the significance of the change in the compression characteristics of a GDL, with respect to its operating temperature, the temperatures selected for the compression tests were 25, 60 and 90 °C.

### 2.2. Experimental Procedures

**Figure 2:**
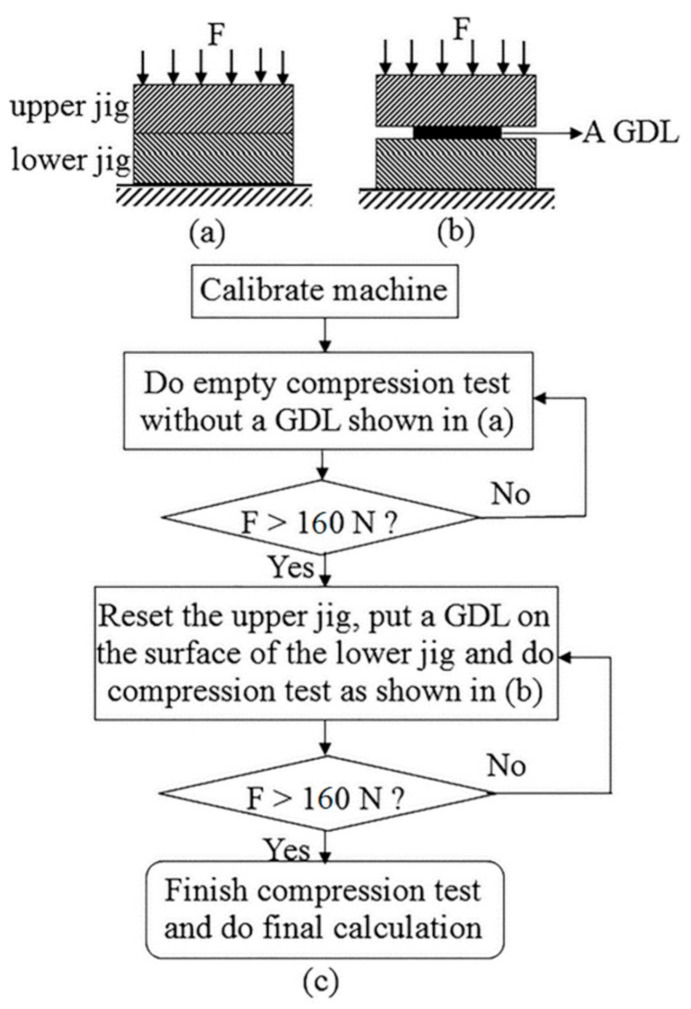
Test schematics (**a**) without and (**b**) with a GDL thin film specimen, and its compression test conducted as, in the chart (**c**).

GDL, a brittle polymer composite in fuel cells, is generally compressed to 0.5–2.5 MPa in between BPPs [23,24]. In terms of material strength, the GDL, which must withstand the assembly pressure of fuel cell stacks, has been reported to bear mechanical loads of up to 10 MPa [25]. Studies are underway to maximize the performance of the fuel cell, so that the level of pressure which the GDL must withstand can be varied, depending on the design. Therefore, for the compression tests to include a full range of mechanical performance of the GDL, the maximum pressure, used in this study, was 10 MPa. In this research, 4 mm × 4 mm square specimens were tailored from a commercial GDL paper, and the load of the tester was set to 160 N to obtain a pressure of 10 MPa. Before the compression test with a specimen was done, the compressive stiffness of the jigs was measured (as shown in Figure 2a). This was done because a load of 10 MPa might cause deformation of the test structure and this might affect the test results. Thus, to determine the net deformation of the GDL specimen, the structural strain of the jigs was subtracted from the total strain tested. While performing each compression test, the specimen was placed on the flat surface of the test jig and compressed by moving the upper jig, as shown in Figure 2b. During the compression test, load and head displacements were automatically recorded by the on-board data acquisition system. Test procedures for thin film specimens were validated to determine the reliable test method, as shown in Figure 2c.

### 2.3. Data Processing

After the thickness of the GDL specimen was measured and the load was recorded, the load versus thickness data were converted into the stress versus strain curves, by taking the area of the specimen and the initial specimen thickness into account. The compression tests were conducted three times at each test temperature (25, 60 and 90 °C).

**Figure 3:**
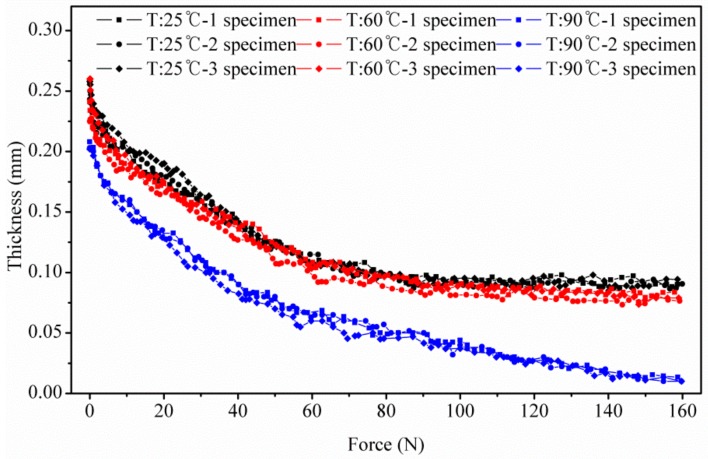
Temperature dependence of GDL’s thickness at compression tests.

**Figure 4:**
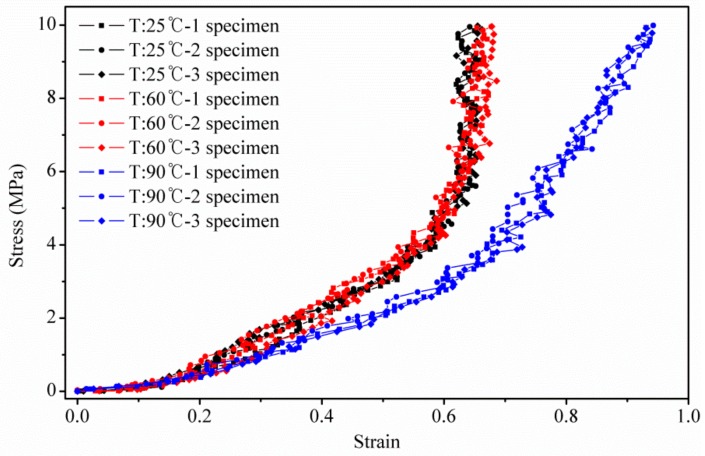
Stress versus strain curves of GDL under different temperatures.

Because of the surface roughness of the thin film specimen GDL, the criterion for measuring the initial thickness of the specimen *t*_0_, should be established. In this test, the specimen thickness that was measured at a compressive load of 5 N (or 0.31 MPa) was considered as the initial thickness *t*_0_. Figure 3 shows the thickness versus load curves and Figure 4 shows the relationship between strain and stress determined accordingly.

(1) $$t_{0}=d_{1@5N}-d_{0@5N}$$

where *d*_1@5N_ is the distance traveled by the upper jig while compressing the specimen at 5 N. *d*_0@5N_ is the distance traveled by the upper jig when compressing the lower jig without the specimen at 5 N. *t_0_* is the initial thickness of the thin film specimen. Similarly, the net specimen thickness is calculated as follows:

(2) $$t=d_{1}-d_{0}$$

where *d*_1_ is the distance traveled by the upper jig during compression of a sample. *d*_0_ is the distance traveled by the upper jig while compressing the lower jig without a sample, and *t* is the net deformation value of the specimen.

Thus, considering the area *A*_0_ and the initial thickness of a specimen, the corresponding strain and stress are calculated by Equations (3) and (4).

(3) $$\varepsilon =\frac{t-t_{0}}{t_{0}}$$

(4) $$\sigma =\frac{F}{A_{0}}$$

where *F* is the compressive force and *A*_0_ is16 mm^2^.

## 3. Results and Discussion

### 3.1. Compression Test Results

The quasi-static compression test was conducted while controlling the load, by moving the head speed of the test machine at 0.01 mm/s. The resolution of the load cell used in the tester was 0.01 N. During the compression test, the instantaneous thickness of the specimen was measured from the movement of the tester head. Furthermore, the magnitude of the corresponding test load was monitored through the load cell. The test was stopped when the compressive load of the tester reached 160 N. As a result, this test yielded the GDL specimens’ thickness versus force curves at the operating temperatures of 25, 60 and 90 °C (see Figure 3). From the graphs obtained at 25, 60 and 90 °C, it was observed that the compressive deformation of all specimens initially reduced steeply in a nonlinear manner as the compressive pressure increased, and then gradually decreased as the compressive load exceeded 60 N, or 37.5 MPa. The main reason for this stiffening phenomenon could be attributed to the reorientation and the breaks of carbon fibers; the broken fibers fill the pores as they are squeezed during compression, as seen from the SEM images. A similar analysis was reported by Kleemann et al. [26], who performed the compression test at 1.8 MPa. The main difference, in this study, is that we considered compression characteristics as a function of temperature and assembly pressure, to study the compression characteristics of GDL. Additionally, we conducted compression tests in a wider range of 0–10 MPa.

The acquired data for thickness versus force were converted into stress versus strain curves, as shown in Figure 4. In Reference [11], although the impact of temperature on GDL’s compressive behavior was discussed, it paid more attention to the GDL’s dynamic compression modulus at the static stress of 3.8 MPa, under a wide temperature range, without sufficiently considering the test load ranges to cover the overall nonlinear properties.

From the stress versus strain curves obtained in this study, it can be seen that the compressive stiffness of the GDL (or the slope of the stress vs. strain curves) was divided into three regions. The compressive stiffness in each region could be considered roughly constant. The first domain had a much lower compression resistance of the GDL, over a very short region, while the GDLs in the remaining two domains, had much greater compressive resistance, spanning over a longer extent. This difference in compressive behavior was directly related to the magnitude of the damage found in the GDL carbon fiber composite, and the change in structural stiffness, due to the high-temperature ductility of the resin, which acted as an adhesive for the carbon fibers in the GDL. In Figure 4, the strain-stress curves, tested at 25 and 60 °C, showed little difference between each other. However, in the tests conducted at 90 °C, the apparent difference in the GDL’s stiffness was strongly influenced by the high-temperature properties of resins or adhesive polymers, which are used in the carbon fiber composites. If a GDL, which has a structurally strong behavior, is required even at high temperatures, the resins or adhesive polymers selected, should have a higher melting temperature.

There are two reasons why the compressive stiffness of the GDL decreased at 90 °C, in this study. The GDL consists of a substrate, an MPL, and a permeable layer between them. The substrate is carbon fiber, bonded with adhesive polymers, such as phenolic resin [27], epoxy resin [28], phenolic resin [29], and cellulose acetate [30]. Of these adhesive polymers, epoxy resins are most often used for GDL fabrication. The mechanical stiffness of almost all of these adhesive resins has an obvious temperature dependence. Agarwal et al. [31] reported that the storage elastic modulus of a rectangular and a single carbon fiber reinforced the epoxy composites decrease, almost linearly, in the temperature range of 80 °C to 110 °C, and stabilizes after 110 °C. The storage elastic modulus of the phenolic resin declines gradually, with increasing temperature, at 100 °C [32]. In addition, the storage modulus of the cellulose acetate reduces slightly with increasing temperature, and then decreases dramatically when the temperature is above 75 °C [30]. Thus, as the foremost reason, it can then be concluded that the storage elastic modulus of these adhesive resins, which are usually used in GDLs, decreases when the temperature is above 60 °C, resulting in a poor compressive stiffness, at high temperatures. This contributes to the difference observed in the GDL’s compressive modulus (or the slope of the strain-stress curves) at 25, 60 and 90 °C. Meanwhile, PTFE, a thermoplastic polymer, is present in the MPL. The melting point of PTFE is high (around 327 °C). However, it shows a strong, temperature dependence on mechanical stiffness, at low temperatures. In particular, the mechanical stiffness of pure PTFE [33] sharply decreases as the temperature increases from –150 °C to 150 °C. Thus, this temperature-sensitive behavior of these adhesive resins and PTFE is believed to directly explain the reduction in compressive stiffness of the GDL, at high temperatures.

### 3.2. GDLs’ Microstructure Observation

**Figure 5:**
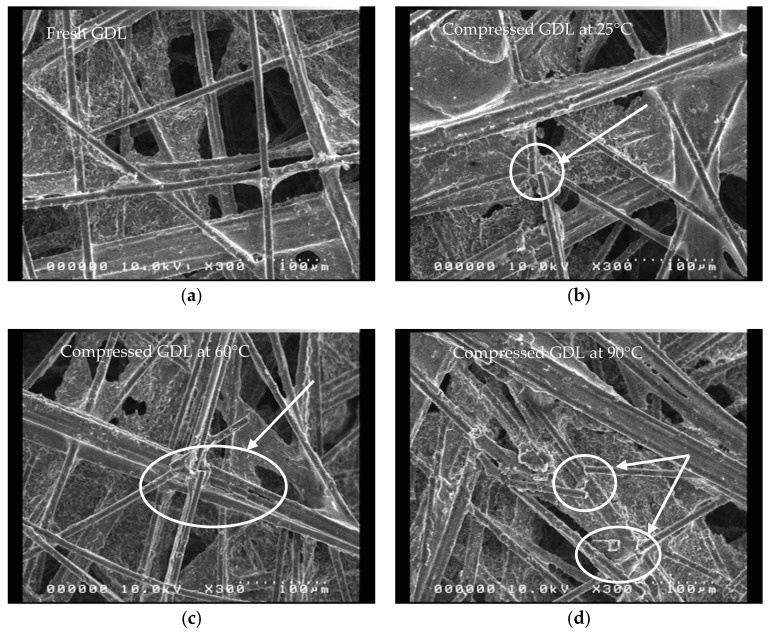
SEM micrographs of (**a**) uncompressed GDL; (**b**) compressed GDL at 25 °C; (**c**) compressed GDL at 60 °C; and (**d**) compressed GDL at 90 °C (Carbon fibers breakage is highlighted with circles as the arrows point).

In order to analyze the damage to the GDL, we compared the microstructure images of the undamaged, and previously tested, GDL specimens. Five SEM images were used for each specimen, to observe the microstructure of the uncompressed GDL and the compressed GDL, at the test temperatures of 25, 60 and 90 °C. The SEM images of these specimens under the same test conditions were observed to be very similar, and each of the representative SEM images was selected only from the uncompressed GDL and the compressed GDL images, at 25, 60 and 90 °C, respectively. It was clearly observed that the image of the uncompressed GDL, as shown in Figure 5a, was composed of a carbon fiber composite, without any damage. However, the carbon fibers of the compressed GDL appeared broken in various forms, due to brittleness. In particular, for the GDLs compressed at 90 °C, it could be observed that the carbon fibers were more severely damaged than those tested at 25 and 60 °C. The greater degree of this damage was due to the softening of the epoxy resin in the GDL, at high temperatures. The degree of damage at 90 °C was different from the cases of the two previous temperatures, and it could be observed that the damage occurred extensively. Additionally, a relative decrease in the percentage of pores was observed in the compressed GDL, at high temperatures.

## 4. Conclusions

In this study, the effect of the operating temperatures on the mechanical properties of a GDL was analyzed. It provides data on an important physical property of GDLs, for its commercial development. The study shows the thermo-mechanical compression characteristics of a commercial GDL, which contributes to the mechanical performance analysis of a fuel cell. With the development of computer analysis technology, the performance of the fuel cell can be verified at the pre-production design stage. The reliability of the result analyses depends on the accuracy of the input properties.

PEMFCs are typically operated at an operating temperature range of −20 °C to 90 °C. This study analyzed the compressive behavior of a commercial GDL, at the operating temperatures of 25, 60 and 90 °C and studied its physical property data. Observing the microstructure of a GDL for test analysis, helps us to understand the process of damage in the GDLs. Therefore, we compared the SEM images for each test condition. The results of this study could be summarized as follows. The operating temperature of a fuel cell is an important factor, which greatly affects the compressive characteristics of the GDL, so, its corresponding mechanical properties should be determined by the operating temperature. In the tests conducted, the GDL showed similar compressive characteristics at the relatively low operating temperatures of 25 and 60 °C. However, at a high operating temperature of 90 °C, the GDL exhibited a very different compressive behavior from the low-temperature cases. The compressive behavior of the GDL, tested at low temperature, was mainly due to the change of microstructure, or the progression of damage in the carbon fibers. However, at a higher temperature, the mechanical properties of the GDL were strongly affected by the temperature-dependent characteristics of the epoxy resin and PTFE, which was observed to have resulted in significantly less resistance to the compression of the GDL, than that observed at lower operating temperatures.

## Figures and Tables

**Table 1:** Properties of JNT 30-A1 GDL^1^.

Property	Value
Area weight	110 ± 5 g/m^2^
Thickness	320 ± 20 μm
Porosity	˂90%
Electrical resistivity	<20 mΩ∙cm^2^

^1^ From JNT Group Company (Hwasung, Korea).

**Table 2:** Specification of material tester BS-205 with an environmental chamber.

Parameter	Value
Maximum temperature	150 °C
Thermostat accuracy	±2 °C
Maximum humidity	90%
Humidity accuracy	±5%
Load cell capacity	200 N
Load cell sensitivity	±0.02 N
Head moving speed	0.001–100 mm/s
